# Evidence for association of autoimmune genes with disabilty in juvenile idiopathic arthritis in a UK cohort

**DOI:** 10.1186/1546-0096-9-S1-P284

**Published:** 2011-09-14

**Authors:** J Cobb, A Hinks, P Martin, E Flynn, R Carrasco, E Baildam, H Foster, J Gardner-Medwin, A Chieng, L Wedderburn, J Davidson, K Hyrich, W Thomson

**Affiliations:** 1 Arthirtis Research UK Epidemiology Unit, The University of Manchester, Manchester, UK; 2 Royal Liverpool Children's Hospitals NHS Trust, Liverpool, UK; 3 Newcastle Hospitals NHS Trust, Newcastle, UK; 4 Royal Hospital for Sick Children, Glasgow, UK; 5 Central Manchester University Hospital NHS Trust, Manchester, UK; 6 Institute of Child Health, UCL, London, UK

## Background

The Childhood Arthritis Prospective Study (CAPS) was initiated to facilitate the investigation of clinical and genetic predictors of long-term outcomes in childhood inflammatory arthritis in UK children.

## Aim

Using 98 candidate loci determined from evidence of previous associations with either JIA susceptibility or other autoimmune diseases, this study aimed to assess the impact of genetics on disease severity and progression. To do this, the childhood health assessment questionnaire (CHAQ) score was used, a key outcome in the measurement of disease activity in JIA patients.

## Methods

Demographic and disease features were collected as part of CAPS at first presentation to paediatric rheumatology, 6 months, and then yearly for 3 years. SNPs were genotyped on a Sequenom MassARRAY^®^ platform. Two analyses were performed to compare the effects of the SNPs on disease severity at presentation to paediatric rheumatology clinics and then the effects over the first 3 years of JIA in a longitudinal logistic regression analysis. Both analyses were undertaken using an additive model of genetic inheritance and adjusting for the key covariates of gender, age at onset and JIA subtype.

## Results

360 children were included in this analysis, with mean age 6.7 ± 4.2 years at disease onset and mean CHAQ score of 0.89 ± 0.77 at first presentation and 0.51 ± 0.64 by 3 years follow-up. 65% of subjects were female, with over 47% presenting with oligoarthritis and 21% with RF negative polyarthritis. A number of interesting findings have emerged, for example the interleukin-2 receptor alpha gene, *IL2RA*, which has previously been associated with JIA susceptibility is now showing evidence of involvement in JIA severity. Of particular interest is that genes which have not previously been associated with JIA susceptibility are showing association with disease severity.

## Conclusion

Findings such as these suggest a genome-wide association study investigating key JIA outcomes is necessary to identify novel disease severity-specific loci for a more comprehensive understanding of the genetics of JIA severity and progression.

